# Determination of Alzheimer's Drugs in a Human Urine Sample by Different Chemometric Methods: Chemometric Determination of Alzheimer's Drugs

**DOI:** 10.1155/2024/5535816

**Published:** 2024-09-27

**Authors:** Güzide Pekcan

**Affiliations:** Department of Chemistry Faculty of Engineering and Natural Sciences Süleyman Demirel University, Isparta 32260, Turkey

**Keywords:** Alzheimer's drug, chemometry, donepezil, rivastigmine

## Abstract

In this study, spectrophotometric determination of donepezil and rivastigmine in healthy human urine samples was carried out by the statistical method. Partial least squares (PLS) and principal component regression (PCR) from multivariate calibration methods were used to evaluate the data obtained from the UV–Vis spectroscopy analysis of the urine sample. Mixtures of each early substance were prepared prior to urine sample analysis, and simultaneous determination of donepezil and rivastigmine was performed on the established chemometric model without any prior separation. The calibration curves of each drug were analyzed, and linearity values were also analyzed. For donepezil and rivastigmine, they were 0.9989 and 0.9997, respectively, and were linear over the concentration range of the synthetic mixture. When both chemometric methods (PLS and PCR) were evaluated in terms of accuracy and reproducibility, very high recoveries and small standard deviations were determined. In the PLS method, the standard error of prediction (SEC), the sum of the prediction residual errors (PRESS), the limit of quantitation (LOQ), and the limit of detection (LOD) values were 0.015, 0.0030, 0.067, 0.24, 0.018, 0.0042, 0.089, and 0.301 for donepezil and rivastigmine, respectively. In the PCR method, SEC, PRESS, LOD, and LOQ values are 0.016, 0.0054, 0.066, and 0.23 for donepezil and 0.022, 0.0062, 0.091, and 0.300 for rivastigmine. Chemometrics is used for speed, simplicity, and reliability. The proposed methods have been successfully applied to a sample of urine.

## 1. Introduction

Alzheimer's disease is a progressive brain ailment that requires ongoing dental treatment to maintain oral health. It gradually loses a person's memory and their capacity to learn, make decisions, communicate, and carry out daily activities [1]. Protein kinases, oxidative stress, and protein aggregates are major factors in the pathophysiology of Alzheimer's disease and may be therapeutic targets [2]. Granular neuropil immunoreactivity to synaptophysin is shown to drop by 50% on average in Alzheimer's disease, indicating a major role for synaptic loss in dementia [3].

Alzheimer's disease medications are used to slow the progression of the disease and improve the patient's quality of life. These drugs can help reduce some symptoms and control some behavioral symptoms. However, no drug has been developed that can completely eradicate the disease once it has started. In the early and middle stages of Alzheimer's disease, medications can improve recovery. In the later stages of the disease, medication is unlikely to be effective. As forgetfulness affects people's daily lives, they should avoid events that make them depressed and have a bad effect. Early diagnosis is very important. Medication and lifestyle changes as soon as possible will slow down the progression of the disease and speed up recovery [4, 5].

Donepezil, rivastigmine, galantamine, and memantine are medicines used for Alzheimer's disease. In this study, the simultaneous determination of the active pharmaceutical ingredients of donepezil and rivastigmine in a human urine sample was carried out. Donepezil inhibits acetylcholinesterase in Alzheimer's disease by preventing the hydrolysis of acetylcholine, thereby reducing the risk of cholinergic neuronal dysfunction [6]. Donepezil improves the main symptoms of Alzheimer's disease and is generally safe and well tolerated [7]. Donepezil is chemically 2,3-dihydro-5,6-dimethoxy-2-[[1-(phenylmethyl)-4-piperidinyl]methly]-1H-inden-1-one hydrochloride [3]. It is an acetyl-cholinesterase inhibitor [8]. Rivastigmine is a brain region–selective acetylcholinesterase inhibitor with a long duration of action that may benefit patients with Alzheimer's disease [9]. Rivastigmine therapy shows a better response in patients with mild to moderate Alzheimer's disease than in those with more severe forms of dementia [10]. The chemical formula of rivastigmine is (S)-3-[(1-dimethylamino)ethyl]-*N*-methylphenyl-carbamate hydrogen tartrate [11].

Chemometrics uses mathematical and software techniques to develop analytical methods and analyze signals and results in pharmaceutical and biomedical analyses [12]. Chemometric methods include partial least squares (PLS) and principal component regression (PCR). These are commonly used in chemical data analysis for predictive modeling [13]. Chemometric methods, such as multivariate data analysis, offer significant advantages to the analytical chemist in the reduction of noise, the handling of interferences, exploratory analysis, and the control of outliers [14]. PLS is a powerful and versatile tool for the analysis of quantitative data in accounting research [15]. PCR has a wider application than previously believed, and response is frequently significantly linked with the main principal components of predictors [16]. Quantitative analysis can be performed with high precision and accuracy on different drug samples without prior separation using chemometric methods such as PCR and PLS [17–20].

The aim of the study was to investigate the challenges of working with human urine samples by means of both classical UV and other instrumental methods. To overcome the complexity of the urine sample, chemometric methods were employed. This study contributes to the existing literature on the subject, which has previously focused on UV spectroscopy and chemometric analysis of drug tablets. The structure of urine is matrix-like, primarily due to the presence of proteins. In order to determine the concentration of drugs in the urine, it was necessary to remove the proteins and apply chemometric calculations using UV spectroscopy.

Alzheimer's drug urine detection is an important area of research, particularly in pharmacokinetic studies, medication adherence monitoring, and understanding drug metabolism. Various analytical techniques are used for detecting Alzheimer's drugs in urine, including chromatography (such as high-performance liquid chromatography [HPLC]), mass spectrometry (MS), immunoassays, and biosensors. Each method has its advantages and limitations in terms of sensitivity, specificity, and ease of use [21–25]. However, chemometric methods were used in addition to the UV method in order to add novelty to the literature and to provide a faster and more cost-effective method compared to the previous methods in the literature. Chemometrics offers advantages in pharmaceutical and biomedical analyses, but validation is crucial for future development and advances [26]. Chemometric methods combined with analytical techniques lead to informative and representative examinations of samples in forensic science [27].

Our biggest aim is to analyze the active ingredients of the drugs used for the treatment of Alzheimer's disease, which is an important place in our daily lives, in human urine samples in terms of health, and to evaluate them with the classical method and to compare them statistically with the classical and newly developed spectrophotometric–chemometric method.

## 2. Materials and Methods

### 2.1. Preparation of the Concentration Set for Donepezil and Rivastigmine

Analytical-grade stock solutions of 100 g/mL donepezil (Sigma-Aldrich, Darmstadt, Germany) and rivastigmine (Sigma-Aldrich, Darmstadt, Germany) were dissolved in 0.1 M HCl. The absorption spectra for donepezil and rivastigmine were recorded between 200 nm and 400 nm using a Shimadzu UV-1700 PharmaSpec spectrophotometer (Kyoto, Japan) connected to a computer running UVProbe software for all measurements and data processing. The training and validation sets consisted of two-component mixtures with varying concentrations. Drug samples containing 7.0 *μ*g/mL and 36.0 *μ*g/mL were dissolved in 25-mL volumetric flasks using 0.1 M HCl. The drugs were administered at different rates in the training and validation sets. For calibration and validation purposes, a total of 10 synthetic combinations were prepared, as shown in Table 1. The calibrating set was constructed using a partial factorial design. Chemometric methods are dependent on a carefully planned experiment. The data were analyzed based on the experimental design, and ten samples were produced. Minitab 17 (İnova, Ankara, Turkey) was used to analyze all concentration and absorbance data and to perform statistical calculations. Minitab is the software for analyzing statistics. In addition to statistical research [28], statistics can be used for learning purposes.

### 2.2. Preparing for Analysis of Human Urine Samples

To prevent the matrix effect, a 20-fold dilution of healthy human urine samples was carried out using deionized water. Subsequently, 2 mL of the diluted urine was added to a tube containing 3 mL of acetonitrile, followed by 4 mL of 10% acetonitrile and various concentrations of pharmaceuticals. The resulting mixture was then combined with 5 mL of urine sample. Once the solutions were prepared, the individual spectra were recorded. It is important to note that the human urine samples were not exposed to drugs at any point in their lifetime. Therefore, a urine sample from children was used. Prior to sample collection, a significant amount of water was consumed. As a result, the urine matrix was prepared appropriately for the analysis of drugs [30].

## 3. Results

**Figure 1:**
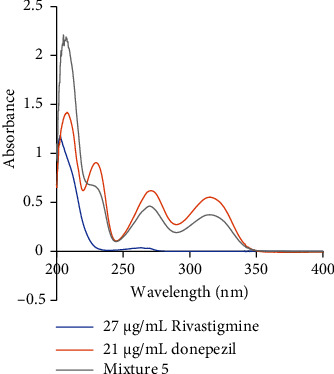
The absorption spectrum of 27 *μ*g/mL rivastigmine, 21 *μ*g/mL donepezil, and Mixture 5.

The absorption spectra of donepezil (maximum wavelength: 230 nm), rivastigmine (maximum wavelength: 203 nm), and the solution of the mixture were obtained over a range of wavelengths.  Figure 1 shows the absorption versus wavelength plots. It can be seen that the absorbance values increase proportionally with increasing concentration when plotting the absorbance versus concentration graphs for three drugs. The fact that the regression coefficient [31] approximates the individual values confirms the linear relationship [32] between absorbance and concentration. In this study, the correlation coefficients for donepezil and rivastigmine are 0.9989 and 0.9997, respectively.

It can be seen that the absorbance values increase proportionally with increasing concentration when plotting the absorbance versus concentration graphs for the three drugs. The fact that the regression coefficient [31] approximates the individual values confirms the linear relationship [32] between absorbance and concentration.

Standard stock solutions containing acetylsalicylic acid, paracetamol, and caffeine were prepared to analyze the basic constituents [33]. The linear working range of the stock solutions was found to be 7–36 *μ*g/mL.

Using PCA, one can minimize the dimensionality of large dimensional data by identifying the dataset with the maximum variance. It identifies the overplanning's general features and offers their dimensional application as well as data availability. The size is definitely the feature to be determined is lost; it is intended to contain little information about these features. This forms most of the features in the plan in a less ostentatious way as “eigenvalues” in order to adapt the future to a modern approach [34]. In the calculation, cross-validation [35, 36] is used to compare the concentrations found with known concentrations. An attempt was made to avoid errors in the calibration of the solutions used in the commercial processing of the samples. To achieve this, a set of concentrations of 10 artificial mixed solutions containing the drugs was prepared. By first examining the pure substances, the spectral range of each component was determined, measuring between 200 and 400 nm. For the set of interval concentrations and in the direction of the statistical program used, the wavelength range was then restricted to 200–400 nm. The absorbance of the spectra recorded in pure form was evaluated during the preparation of the concentration set.

### 3.1. PCR and PLS

The chemometric methods that were used in this study were the PLS and the PCR. PLS [37, 38] and PCR [39, 40] are multivariate calibration methods with many advantages over the full spectrum and have been successfully applied to the spectrophotometric analysis of multicomponent mixtures. PLS is based on spectral changes. It does not depend on the concentration of the components. In the PLS method, the spectral decomposition is weighted according to the concentration of the components. There is a significant difference in the predictive ability of these two approaches, with PLS appearing to be more predictive than PCR methods [41].

The statistical parameters used for the validation of donepezil and rivastigmine calibrations have been found to confirm the validity of the PLS and PCR methods. The results presented in Table 2 demonstrate high accuracy, with low standard deviations and recoveries close to 100%, indicating reasonable results.

### 3.2. The Method's Validation

The chemometric approach was validated for linearity, accuracy, intraday and interday precision, limit of detection (LOD), and limit of quantitation (LOQ) in accordance with the criteria set by the International Council for Harmonisation (ICH) [42–44]. The calibration utilized the sum of the prediction residual errors (PRESS) [45], which is calculated as follows:

(1) $$\begin{array}{c} \text{PRESS}=\overset{n}{\underset{i=1}{\sum }}{\left({С}_{i}^{\text{added}}-{С}_{i}^{\text{found}}\right)}^{2}, \end{array}$$

where *C*_*i*_^added^ is the actual concentration, the added concentration of the drug, and *C*_*i*_^found^ is the predicted concentration, the calculated concentration of the drug. Based on the actual and anticipated concentrations of the samples, the PRESS values for rivastigmine and donepezil were determined (Table 3).

It is worth noting that normalizing PRESS values using this method may not be entirely accurate if the datasets being compared do not have the same number of samples. In addition, it is important to mention that the number of samples is included in the standard error of prediction (SEC), as shown in equation (2). A few statistical criteria were used to determine whether or not the calibration worked.

The SEC has been calculated in accordance with the following formula:

(2) $$\begin{array}{c} \text{SEC}=\sqrt{\frac{\sum_{i=1}^{n}{\left(C_{i}^{\text{added}}-C_{i}^{\text{found}}\right)}^{2}}{n-1}}, \end{array}$$

where *n* is the total number of synthetic mixtures.

As shown in equation (3), another validation parameter is RMSEC [46], which is calculated as

(3) $$\begin{array}{c} \text{RMSEC}=\sqrt{\frac{\text{PRESS}}{n}}. \end{array}$$

The LOQ and LOD are linked but have different definitions. The LOQ and LOD have different definitions as shown in the following equations [47]:

(4) $$\begin{array}{c} \text{LOD}=\frac{3\text{Sa}}{m}, \end{array}$$

(5) $$\begin{array}{c} \text{LOQ}=\frac{10\text{Sa}}{m}, \end{array}$$

where *m* is the slope and Sa is the value of the adjusted standard deviation.

In evaluating the calculated LOD values, LOQ > LOD and LOQ = LOD were considered [48].

The values of PRESS and SEC are both close to zero, which suggests an improvement in accuracy. It is worth noting that Table 3 shows that the calculated values of PRESS and SEC are also close to zero for both the PLS and PCR methods.

The UV spectrophotometric method was utilized to analyze the materials, and Snedecor's *F*-test [49] was employed to assess the effectiveness of the investigated chemometric procedures. The differences between the single-use tests were analyzed using the ANOVA method on real samples for each drug. The *F*-values of the Snedecor test were calculated and compared with the experimental *F*-values in this study, using the same mathematical procedure for each drug. The *F*-values obtained from the experiment did not surpass the *F*-value from the variance analysis, which was 4.13 with a 95% confidence interval. The *F*-test values for donepezil and rivastigmine were calculated using the PLS technique, resulting in values of 0.00065 and 0.00068, respectively, both with a *p* value of 0.95. The *F*-test value for donepezil was calculated as 0.00072 with a *p* value of 0.95, and for rivastigmine, it was calculated as 0.00075. Based on the PCR technique, these techniques were found to be significantly different.

### 3.3. Analysis of a Sample of Human Urine

The experimental values of the PCR and PLS methods for human urine samples are shown in Table 4. It can be seen that the results obtained are very close to each other. Chemometric methods have been successfully applied to the determination of drugs in healthy human urine samples and have produced highly accurate results, as shown in Table 4. The statistical values obtained appear to be adequate for the simultaneous detection of these substances in human urine samples. The unadulterated urine sample does not contain any amount of medication, including rivastigmine and donepezil.

## 4. Discussion

The use of PLS and PCR proved effective in determining drug concentrations in synthetic solutions. Our findings indicated minimal prediction errors and high correlation coefficients, highlighting a robust linear relationship between expected and actual concentrations (Table 3). Moreover, these methods demonstrated predictive ability when applied to binary mixtures and component concentration ratios.

In our study, chemometric analysis of UV spectroscopy data was employed to determine the purity of drug compounds in a sample containing two different active ingredients. Initially, UV spectra of donepezil and rivastigmine were recorded to establish the required purity levels for the study. Subsequently, we conducted analytical studies wherein the UV spectra of donepezil and rivastigmine were statistically developed by our method. Standard curves were then subjected to regression analysis, and the resulting data were statistically evaluated.

The *F*-test was utilized to analyze the data obtained with the chemometric program. A comparison was made between the results and the synthetic model created during the experimental design phase before analyzing the effervescent tablet sample. Encouragingly, the synthetic models exhibited agreement with the experimental results obtained from the chemometric program when applied to the drug sample mixture. Consequently, the model based on the *F*-test results was deemed applicable to the drug sample mixture. The *F*-test values for donepezil and rivastigmine were calculated using the PLS technique, resulting in values of 0.00065 and 0.00068, respectively, both with a *p* value of 0.95. The *F*-test value for donepezil was calculated as 0.00072 with a *p* value of 0.95, and for rivastigmine, it was calculated as 0.00075.

The application of chemometrics, which is the application of software, mathematics, and statistics to chemistry, to the simultaneous determination of the active substances ezetimibe and simvastatin used for the treatment of hyperlipidemia, and to develop a new method that is alternative, faster, and cost-effective than the classical methods previously used in the pharmaceutical industry with the chemometrics program was one of the contributions of this study to the literature. No studies on chemometric determination of Alzheimer's drugs in urine samples were found in the literature [21–25]. Chemometric techniques allow even complex systems to be analyzed spectrophotometrically as is, without any preseparation, and this convenience has been used for the simultaneous determination of drug-active ingredients used in the treatment of Alzheimer's disease and statistically more reliable results have been obtained (Table 4). By going through an accurate experimental design with the chemometrics method, the loss of time and work lost by the trial and error method is prevented.

## 5. Conclusion

The proteins were precipitated from the urine and measured in the UV–visible range by means of specific procedures. The results of the study were high recoveries, an indication that the drugs were not bound to the urine proteins. As part of the validation process, errors were predicted for mixtures containing both donepezil and rivastigmine. In addition, the sum of squares (PRESS) and standard error (SEC) of the calibration were calculated and reduced to zero, indicating a high level of accuracy in the results. Drug samples and human urine were analyzed using chemometric methods. Reproducible results were obtained due to the high sensitivity of the method. On the basis of the results obtained, it appears that this method may be suitable for the simultaneous determination of donepezil and rivastigmine in human urine.

## Figures and Tables

**Table 1:** Concentration set for donepezil and rivastigmine [29].

**No.**	**Donepezil (*μ*g/mL)**	**Rivastigmine (*μ*g/mL)**
1	7	9
2	7	18
3	7	36
4	14	9
5	14	18
6	14	36
7	27	9
8	27	18
9	27	27
10	27	9

**Table 2:** Summary of recovery results obtained in synthetic mixtures for PLS and PCR methods [29].

**No.**	**Donepezil recovery (%)**		**Rivastigmine recovery (%)**	
	**PLS**	**PCR**	**PLS**	**PCR**
1	99.85	99.57	100.11	98.78
2	98.14	90.28	99.83	99.17
3	98.85	97.85	98.77	100.02
4	99.71	99.21	99.77	100.00
5	98.85	99.57	99.83	99.39
6	99.42	99.79	99.61	99.64
7	99.81	99.85	99.55	98.22
8	95.88	99.52	99.66	98.78
9	97.96	97.15	99.51	99.22
10	99.78	99.79	99.44	99.33
Mean	98.83	99.26	99.61	99.26
RSD%	1.26	2.97	0.35	0.56

**Table 3:** Statistical parameter values for calibration of step-simultaneous donepezil and rivastigmine determinations by means of PLS and PCR techniques.

**Parameters**	**Method**	**Donepezil**	**Rivastigmine**
SEC	PLS	0.015	0.018
	PCR	0.016	0.022

PRESS	PLS	0.0030	0.0042
	PCR	0.0054	0.0062

RMSEC	PLS	0.0594	0.0589
	PCR	0.0497	0.0462

LOD (*μ*g/mL)	PLS	0.067	0.089
	PCR	0.066	0.091

LOQ (*μ*g/mL)	PLS	0.24	0.301
	PCR	0.23	0.300

Accuracy (%) (recovery ± SD)	PLS	98.83 ± 1.26	99.61 ± 0.35
	PCR	99.26 ± 2.97	99.26 ± 0.56

Precision (reproducibility)			
Intraday (%) (recovery ± SD) (*n*:6)	PLS	98.87 ± 0.97	98.62 ± 0.45
	PCR	97.89 ± 0.56	98.56 ± 0.67

Interday (%) (recovery ± SD) (*n*:6)	PLS	99.81 ± 0.58	97.96 ± 0.75
	PCR	98.52 ± 0.32	98.69 ± 0.69

**Table 4:** Determination of donepezil and rivastigmine in human urine using PLS and PCR techniques.

**Mix no.**	**Added (mg/L)**	**Found (mg/L)**	**Recovery (% mean)**	**Added (mg/L)**	**Found (mg/L)**	**Recovery (% mean)**
	Donepezil (PLS)			Rivastigmine (PLS)		

1	7	6.89	98.43	9	8.98	99.78
2	14	14.01	100.07	18	17.96	99.77
3	21	20.95	99.76	27	26.68	98.81
4	28	27.95	99.82	28	27.92	99.71
5	35	34.97	99.91	35	34.95	99.86
Mean ± SD			99.60 ± 0.66			99.59 ± 0.44

	Donepezil (PCR)			Rivastigmine (PCR)		

1	7	6.96	99.42	9	8.95	99.44
2	14	13.95	99.64	18	17.88	99.33
3	21	20.98	99.9	27	26.94	99.78
4	28	27.88	99.57	28	27.93	99.75
5	35	34.96	99.89	35	34.97	99.91
Mean ± SD			99.64 ± 0.21			99.64 ± 0.26

## Data Availability

The data that support the findings of this study are available from the corresponding author upon reasonable request.
